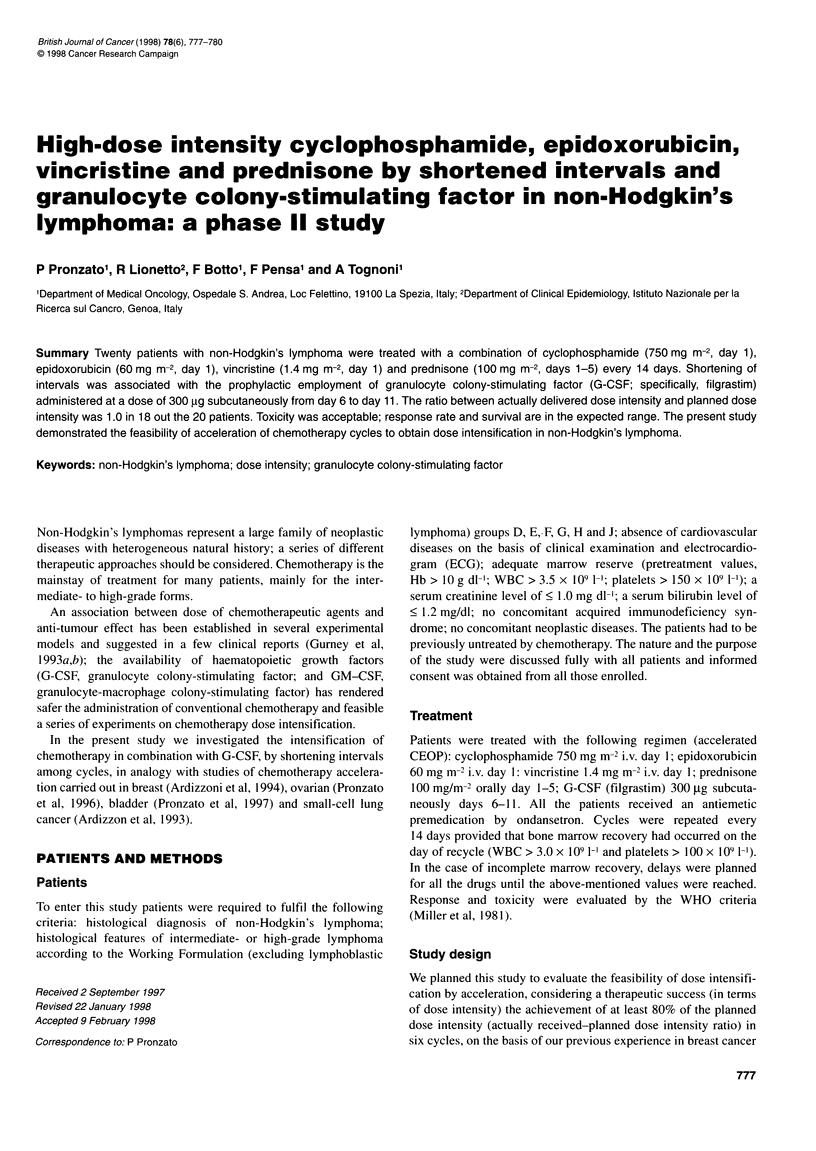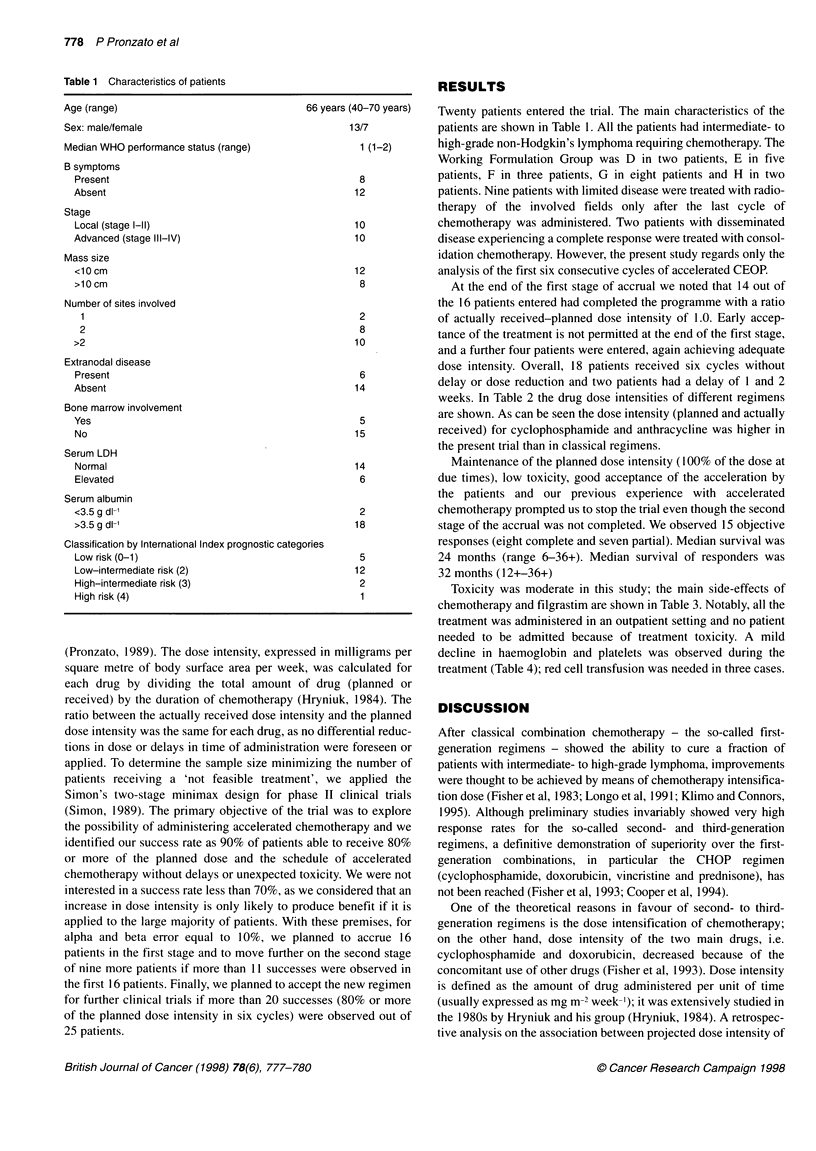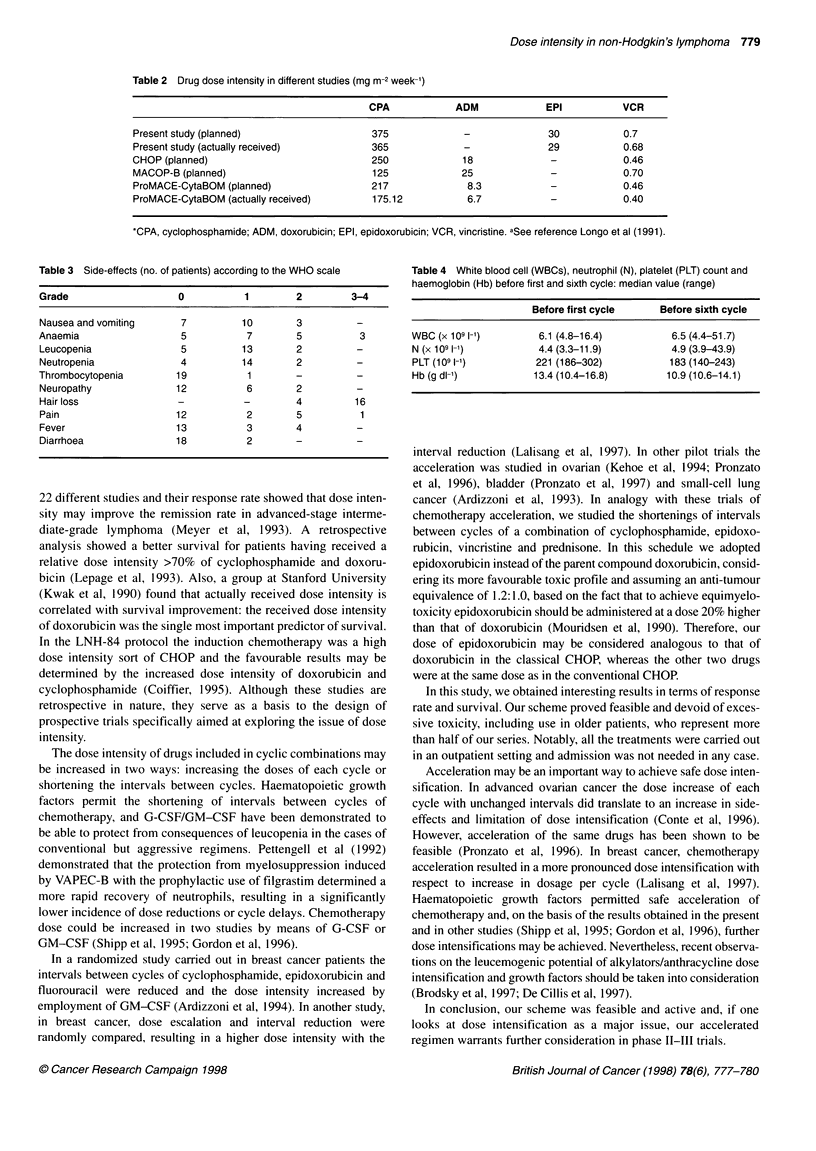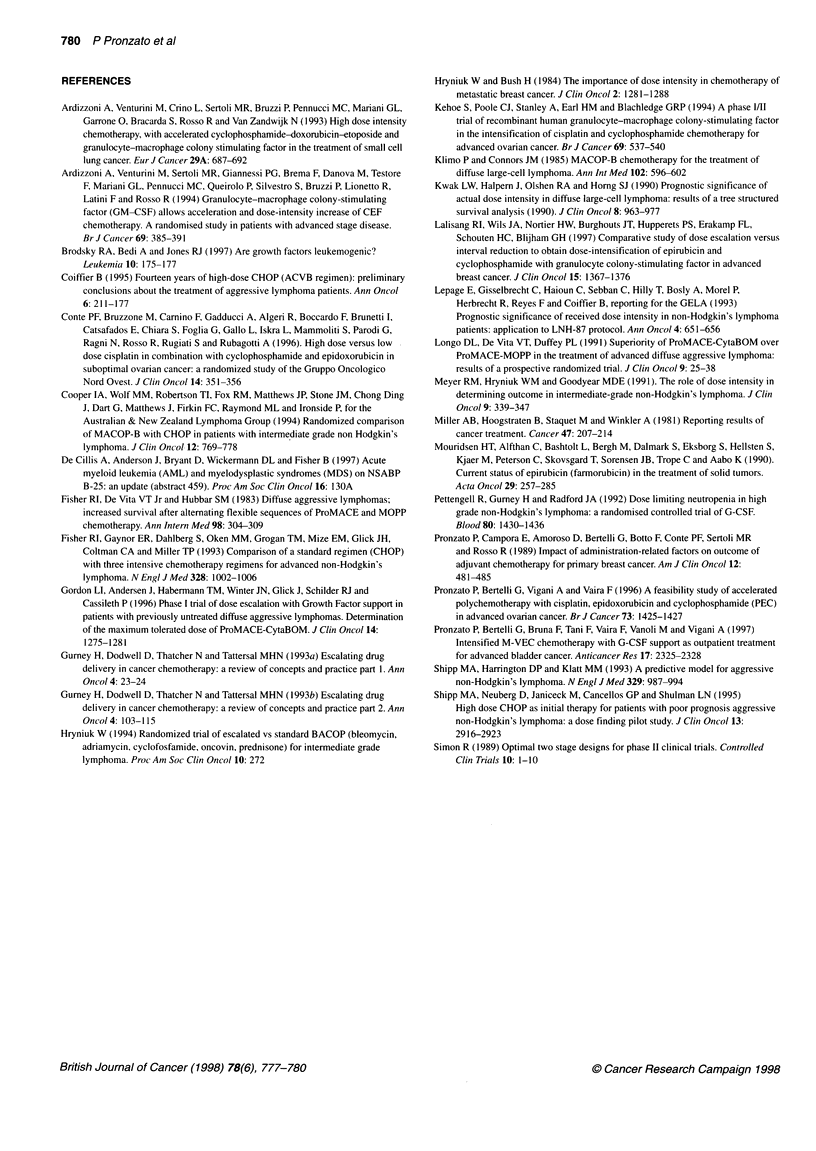# High-dose intensity cyclophosphamide, epidoxorubicin, vincristine and prednisone by shortened intervals and granulocyte colony-stimulating factor in non-Hodgkin's lymphoma: a phase II study.

**DOI:** 10.1038/bjc.1998.578

**Published:** 1998-09

**Authors:** P. Pronzato, R. Lionetto, F. Botto, F. Pensa, A. Tognoni

**Affiliations:** Department of Medical Oncology, Ospedale S. Andrea, Loc Felettino, La Spezia, Italy.

## Abstract

Twenty patients with non-Hodgkin's lymphoma were treated with a combination of cyclophosphamide (750 mg m(-2), day 1), epidoxorubicin (60 mg m(-2), day 1), vincristine (1.4 mg m(-2), day 1) and prednisone (100 mg m(-2), days 1-5) every 14 days. Shortening of intervals was associated with the prophylactic employment of granulocyte colony-stimulating factor (G-CSF; specifically, filgrastim) administered at a dose of 300 microg subcutaneously from day 6 to day 11. The ratio between actually delivered dose intensity and planned dose intensity was 1.0 in 18 out the 20 patients. Toxicity was acceptable; response rate and survival are in the expected range. The present study demonstrated the feasibility of acceleration of chemotherapy cycles to obtain dose intensification in non-Hodgkin's lymphoma.


British Journal of Cancer (1998) 78(6), 777–780
© 1998 Cancer Research Campaign

### High-dose intensity cyclophosphamide, epidoxorubicin, vincristine and prednisone by shortened intervals and granulocyte colony-stimulating factor in non-Hodgkin's lymphoma: a phase II study

**P Pronzato[1], R Lionetto[2], F Botto[1], F Pensa[1] and A Tognoni[1]**

[1]Department of Medical Oncology, Ospedale S. Andrea, Loc Felettino, 19100 La Spezia, Italy; [2]Department of Clinical Epidemiology, Istituto Nazionale per la Ricerca sul Cancro, Genoa, Italy

**Summary** Twenty patients with non-Hodgkin's lymphoma were treated with a combination of cyclophosphamide (750 mg m$^{-2}$, day 1), epidoxorubicin (60 mg m$^{-2}$, day 1), vincristine (1.4 mg m$^{-2}$, day 1) and prednisone (100 mg m$^{-2}$, days 1–5) every 14 days. Shortening of intervals was associated with the prophylactic employment of granulocyte colony-stimulating factor (G-CSF; specifically, filgrastim) administered at a dose of 300 μg subcutaneously from day 6 to day 11. The ratio between actually delivered dose intensity and planned dose intensity was 1.0 in 18 out the 20 patients. Toxicity was acceptable; response rate and survival are in the expected range. The present study demonstrated the feasibility of acceleration of chemotherapy cycles to obtain dose intensification in non-Hodgkin's lymphoma.

**Keywords:** non-Hodgkin's lymphoma; dose intensity; granulocyte colony-stimulating factor

Non-Hodgkin's lymphomas represent a large family of neoplastic diseases with heterogeneous natural history; a series of different therapeutic approaches should be considered. Chemotherapy is the mainstay of treatment for many patients, mainly for the intermediate- to high-grade forms.

An association between dose of chemotherapeutic agents and anti-tumour effect has been established in several experimental models and suggested in a few clinical reports (Gurney et al, 1993*a*,*b*); the availability of haematopoietic growth factors (G-CSF, granulocyte colony-stimulating factor; and GM–CSF, granulocyte-macrophage colony-stimulating factor) has rendered safer the administration of conventional chemotherapy and feasible a series of experiments on chemotherapy dose intensification.

In the present study we investigated the intensification of chemotherapy in combination with G-CSF, by shortening intervals among cycles, in analogy with studies of chemotherapy acceleration carried out in breast (Ardizzoni et al, 1994), ovarian (Pronzato et al, 1996), bladder (Pronzato et al, 1997) and small-cell lung cancer (Ardizzon et al, 1993).

### PATIENTS AND METHODS

### Patients

To enter this study patients were required to fulfil the following criteria: histological diagnosis of non-Hodgkin's lymphoma; histological features of intermediate- or high-grade lymphoma according to the Working Formulation (excluding lymphoblastic lymphoma) groups D, E, F, G, H and J; absence of cardiovascular diseases on the basis of clinical examination and electrocardiogram (ECG); adequate marrow reserve (pretreatment values, Hb > 10 g dl$^{-1}$; WBC > 3.5 × 10$^9$ l$^{-1}$; platelets > 150 × 10$^9$ l$^{-1}$); a serum creatinine level of ≤ 1.0 mg dl$^{-1}$; a serum bilirubin level of ≤ 1.2 mg/dl; no concomitant acquired immunodeficiency syndrome; no concomitant neoplastic diseases. The patients had to be previously untreated by chemotherapy. The nature and the purpose of the study were discussed fully with all patients and informed consent was obtained from all those enrolled.

### Treatment

Patients were treated with the following regimen (accelerated CEOP): cyclophosphamide 750 mg m$^{-2}$ i.v. day 1; epidoxorubicin 60 mg m$^{-2}$ i.v. day 1: vincristine 1.4 mg m$^{-2}$ i.v. day 1; prednisone 100 mg/m$^{-2}$ orally day 1–5; G-CSF (filgrastim) 300 μg subcutaneously days 6–11. All the patients received an antiemetic premedication by ondansetron. Cycles were repeated every 14 days provided that bone marrow recovery had occurred on the day of recycle (WBC > 3.0 × 10$^9$ l$^{-1}$ and platelets > 100 × 10$^9$ l$^{-1}$). In the case of incomplete marrow recovery, delays were planned for all the drugs until the above-mentioned values were reached. Response and toxicity were evaluated by the WHO criteria (Miller et al, 1981).

### Study design

We planned this study to evaluate the feasibility of dose intensification by acceleration, considering a therapeutic success (in terms of dose intensity) the achievement of at least 80% of the planned dose intensity (actually received–planned dose intensity ratio) in six cycles, on the basis of our previous experience in breast cancer

*Received 2 September 1997*
*Revised 22 January 1998*
*Accepted 9 February 1998*

*Correspondence to:* P Pronzato

**Table 1** Characteristics of patients

| | |
|---|---|
| Age (range) | 66 years (40–70 years) |
| Sex: male/female | 13/7 |
| Median WHO performance status (range) | 1 (1–2) |
| B symptoms | |
| Present | 8 |
| Absent | 12 |
| Stage | |
| Local (stage I–II) | 10 |
| Advanced (stage III–IV) | 10 |
| Mass size | |
| <10 cm | 12 |
| >10 cm | 8 |
| Number of sites involved | |
| 1 | 2 |
| 2 | 8 |
| >2 | 10 |
| Extranodal disease | |
| Present | 6 |
| Absent | 14 |
| Bone marrow involvement | |
| Yes | 5 |
| No | 15 |
| Serum LDH | |
| Normal | 14 |
| Elevated | 6 |
| Serum albumin | |
| <3.5 g $dl^{-1}$ | 2 |
| >3.5 g $dl^{-1}$ | 18 |
| Classification by International Index prognostic categories | |
| Low risk (0–1) | 5 |
| Low–intermediate risk (2) | 12 |
| High–intermediate risk (3) | 2 |
| High risk (4) | 1 |

(Pronzato, 1989). The dose intensity, expressed in milligrams per square metre of body surface area per week, was calculated for each drug by dividing the total amount of drug (planned or received) by the duration of chemotherapy (Hryniuk, 1984). The ratio between the actually received dose intensity and the planned dose intensity was the same for each drug, as no differential reductions in dose or delays in time of administration were foreseen or applied. To determine the sample size minimizing the number of patients receiving a 'not feasible treatment', we applied the Simon's two-stage minimax design for phase II clinical trials (Simon, 1989). The primary objective of the trial was to explore the possibility of administering accelerated chemotherapy and we identified our success rate as 90% of patients able to receive 80% or more of the planned dose and the schedule of accelerated chemotherapy without delays or unexpected toxicity. We were not interested in a success rate less than 70%, as we considered that an increase in dose intensity is only likely to produce benefit if it is applied to the large majority of patients. With these premises, for alpha and beta error equal to 10%, we planned to accrue 16 patients in the first stage and to move further on the second stage of nine more patients if more than 11 successes were observed in the first 16 patients. Finally, we planned to accept the new regimen for further clinical trials if more than 20 successes (80% or more of the planned dose intensity in six cycles) were observed out of 25 patients.

### RESULTS

Twenty patients entered the trial. The main characteristics of the patients are shown in Table 1. All the patients had intermediate- to high-grade non-Hodgkin's lymphoma requiring chemotherapy. The Working Formulation Group was D in two patients, E in five patients, F in three patients, G in eight patients and H in two patients. Nine patients with limited disease were treated with radiotherapy of the involved fields only after the last cycle of chemotherapy was administered. Two patients with disseminated disease experiencing a complete response were treated with consolidation chemotherapy. However, the present study regards only the analysis of the first six consecutive cycles of accelerated CEOP.

At the end of the first stage of accrual we noted that 14 out of the 16 patients entered had completed the programme with a ratio of actually received–planned dose intensity of 1.0. Early acceptance of the treatment is not permitted at the end of the first stage, and a further four patients were entered, again achieving adequate dose intensity. Overall, 18 patients received six cycles without delay or dose reduction and two patients had a delay of 1 and 2 weeks. In Table 2 the drug dose intensities of different regimens are shown. As can be seen the dose intensity (planned and actually received) for cyclophosphamide and anthracycline was higher in the present trial than in classical regimens.

Maintenance of the planned dose intensity (100% of the dose at due times), low toxicity, good acceptance of the acceleration by the patients and our previous experience with accelerated chemotherapy prompted us to stop the trial even though the second stage of the accrual was not completed. We observed 15 objective responses (eight complete and seven partial). Median survival was 24 months (range 6–36+). Median survival of responders was 32 months (12+–36+)

Toxicity was moderate in this study; the main side-effects of chemotherapy and filgrastim are shown in Table 3. Notably, all the treatment was administered in an outpatient setting and no patient needed to be admitted because of treatment toxicity. A mild decline in haemoglobin and platelets was observed during the treatment (Table 4); red cell transfusion was needed in three cases.

### DISCUSSION

After classical combination chemotherapy – the so-called first-generation regimens – showed the ability to cure a fraction of patients with intermediate- to high-grade lymphoma, improvements were thought to be achieved by means of chemotherapy intensification dose (Fisher et al, 1983; Longo et al, 1991; Klimo and Connors, 1995). Although preliminary studies invariably showed very high response rates for the so-called second- and third-generation regimens, a definitive demonstration of superiority over the first-generation combinations, in particular the CHOP regimen (cyclophosphamide, doxorubicin, vincristine and prednisone), has not been reached (Fisher et al, 1993; Cooper et al, 1994).

One of the theoretical reasons in favour of second- to third-generation regimens is the dose intensification of chemotherapy; on the other hand, dose intensity of the two main drugs, i.e. cyclophosphamide and doxorubicin, decreased because of the concomitant use of other drugs (Fisher et al, 1993). Dose intensity is defined as the amount of drug administered per unit of time (usually expressed as mg $m^{-2}$ $week^{-1}$); it was extensively studied in the 1980s by Hryniuk and his group (Hryniuk, 1984). A retrospective analysis on the association between projected dose intensity of

© Cancer Research Campaign 1998

**Table 2** Drug dose intensity in different studies (mg m$^{-2}$ week$^{-1}$)

| | CPA | ADM | EPI | VCR |
|---|---|---|---|---|
| Present study (planned) | 375 | – | 30 | 0.7 |
| Present study (actually received) | 365 | – | 29 | 0.68 |
| CHOP (planned) | 250 | 18 | – | 0.46 |
| MACOP-B (planned) | 125 | 25 | – | 0.70 |
| ProMACE-CytaBOM (planned) | 217 | 8.3 | – | 0.46 |
| ProMACE-CytaBOM (actually received) | 175.12 | 6.7 | – | 0.40 |

*CPA, cyclophosphamide; ADM, doxorubicin; EPI, epidoxorubicin; VCR, vincristine. [a]See reference Longo et al (1991).

**Table 3** Side-effects (no. of patients) according to the WHO scale

| Grade | 0 | 1 | 2 | 3–4 |
|---|---|---|---|---|
| Nausea and vomiting | 7 | 10 | 3 | – |
| Anaemia | 5 | 7 | 5 | 3 |
| Leucopenia | 5 | 13 | 2 | – |
| Neutropenia | 4 | 14 | 2 | – |
| Thrombocytopenia | 19 | 1 | – | – |
| Neuropathy | 12 | 6 | 2 | – |
| Hair loss | – | – | 4 | 16 |
| Pain | 12 | 2 | 5 | 1 |
| Fever | 13 | 3 | 4 | – |
| Diarrhoea | 18 | 2 | – | – |

**Table 4** White blood cell (WBCs), neutrophil (N), platelet (PLT) count and haemoglobin (Hb) before first and sixth cycle: median value (range)

| | Before first cycle | Before sixth cycle |
|---|---|---|
| WBC (× $10^9$ $l^{-1}$) | 6.1 (4.8–16.4) | 6.5 (4.4–51.7) |
| N (× $10^9$ $l^{-1}$) | 4.4 (3.3–11.9) | 4.9 (3.9–43.9) |
| PLT ($10^9$ $l^{-1}$) | 221 (186–302) | 183 (140–243) |
| Hb (g $dl^{-1}$) | 13.4 (10.4–16.8) | 10.9 (10.6–14.1) |

22 different studies and their response rate showed that dose intensity may improve the remission rate in advanced-stage intermediate-grade lymphoma (Meyer et al, 1993). A retrospective analysis showed a better survival for patients having received a relative dose intensity >70% of cyclophosphamide and doxorubicin (Lepage et al, 1993). Also, a group at Stanford University (Kwak et al, 1990) found that actually received dose intensity is correlated with survival improvement: the received dose intensity of doxorubicin was the single most important predictor of survival. In the LNH-84 protocol the induction chemotherapy was a high dose intensity sort of CHOP and the favourable results may be determined by the increased dose intensity of doxorubicin and cyclophosphamide (Coiffier, 1995). Although these studies are retrospective in nature, they serve as a basis to the design of prospective trials specifically aimed at exploring the issue of dose intensity.

The dose intensity of drugs included in cyclic combinations may be increased in two ways: increasing the doses of each cycle or shortening the intervals between cycles. Haematopoietic growth factors permit the shortening of intervals between cycles of chemotherapy, and G-CSF/GM–CSF have been demonstrated to be able to protect from consequences of leucopenia in the cases of conventional but aggressive regimens. Pettengell et al (1992) demonstrated that the protection from myelosuppression induced by VAPEC-B with the prophylactic use of filgrastim determined a more rapid recovery of neutrophils, resulting in a significantly lower incidence of dose reductions or cycle delays. Chemotherapy dose could be increased in two studies by means of G-CSF or GM–CSF (Shipp et al, 1995; Gordon et al, 1996).

In a randomized study carried out in breast cancer patients the intervals between cycles of cyclophosphamide, epidoxorubicin and fluorouracil were reduced and the dose intensity increased by employment of GM–CSF (Ardizzoni et al, 1994). In another study, in breast cancer, dose escalation and interval reduction were randomly compared, resulting in a higher dose intensity with the interval reduction (Lalisang et al, 1997). In other pilot trials the acceleration was studied in ovarian (Kehoe et al, 1994; Pronzato et al, 1996), bladder (Pronzato et al, 1997) and small-cell lung cancer (Ardizzoni et al, 1993). In analogy with these trials of chemotherapy acceleration, we studied the shortenings of intervals between cycles of a combination of cyclophosphamide, epidoxorubicin, vincristine and prednisone. In this schedule we adopted epidoxorubicin instead of the parent compound doxorubicin, considering its more favourable toxic profile and assuming an anti-tumour equivalence of 1.2:1.0, based on the fact that to achieve equimyelotoxicity epidoxorubicin should be administered at a dose 20% higher than that of doxorubicin (Mouridsen et al, 1990). Therefore, our dose of epidoxorubicin may be considered analogous to that of doxorubicin in the classical CHOP, whereas the other two drugs were at the same dose as in the conventional CHOP.

In this study, we obtained interesting results in terms of response rate and survival. Our scheme proved feasible and devoid of excessive toxicity, including use in older patients, who represent more than half of our series. Notably, all the treatments were carried out in an outpatient setting and admission was not needed in any case.

Acceleration may be an important way to achieve safe dose intensification. In advanced ovarian cancer the dose increase of each cycle with unchanged intervals did translate to an increase in side-effects and limitation of dose intensification (Conte et al, 1996). However, acceleration of the same drugs has been shown to be feasible (Pronzato et al, 1996). In breast cancer, chemotherapy acceleration resulted in a more pronounced dose intensification with respect to increase in dosage per cycle (Lalisang et al, 1997). Haematopoietic growth factors permitted safe acceleration of chemotherapy and, on the basis of the results obtained in the present and in other studies (Shipp et al, 1995; Gordon et al, 1996), further dose intensifications may be achieved. Nevertheless, recent observations on the leucemogenic potential of alkylators/anthracycline dose intensification and growth factors should be taken into consideration (Brodsky et al, 1997; De Cillis et al, 1997).

In conclusion, our scheme was feasible and active and, if one looks at dose intensification as a major issue, our accelerated regimen warrants further consideration in phase II–III trials.

© Cancer Research Campaign 1998

### REFERENCES

Ardizzoni A, Venturini M, Crino L, Sertoli MR, Bruzzi P, Pennucci MC, Mariani GL, Garrone O, Bracarda S, Rosso R and Van Zandwijk N (1993) High dose intensity chemotherapy, with accelerated cyclophosphamide–doxorubicin–etoposide and granulocyte–macrophage colony stimulating factor in the treatment of small cell lung cancer. *Eur J Cancer* **29A**: 687–692

Ardizzoni A, Venturini M, Sertoli MR, Giannessi PG, Brema F, Danova M, Testore F, Mariani GL, Pennucci MC, Queirolo P, Silvestro S, Bruzzi P, Lionetto R, Latini F and Rosso R (1994) Granulocyte–macrophage colony-stimulating factor (GM–CSF) allows acceleration and dose-intensity increase of CEF chemotherapy. A randomised study in patients with advanced stage disease. *Br J Cancer* **69**: 385–391

Brodsky RA, Bedi A and Jones RJ (1997) Are growth factors leukemogenic? *Leukemia* **10**: 175–177

Coiffier B (1995) Fourteen years of high-dose CHOP (ACVB regimen): preliminary conclusions about the treatment of aggressive lymphoma patients. *Ann Oncol* **6**: 211–177

Conte PF, Bruzzone M, Carnino F, Gadducci A, Algeri R, Boccardo F, Brunetti I, Catsafados E, Chiara S, Foglia G, Gallo L, Iskra L, Mammoliti S, Parodi G, Ragni N, Rosso R, Rugiati S and Rubagotti A (1996). High dose versus low dose cisplatin in combination with cyclophosphamide and epidoxorubicin in suboptimal ovarian cancer: a randomized study of the Gruppo Oncologico Nord Ovest. *J Clin Oncol* **14**: 351–356

Cooper IA, Wolf MM, Robertson TI, Fox RM, Matthews JP, Stone JM, Chong Ding J, Dart G, Matthews J, Firkin FC, Raymond ML and Ironside P, for the Australian & New Zealand Lymphoma Group (1994) Randomized comparison of MACOP-B with CHOP in patients with intermediate grade non Hodgkin's lymphoma. *J Clin Oncol* **12**: 769–778

De Cillis A, Anderson J, Bryant D, Wickermann DL and Fisher B (1997) Acute myeloid leukemia (AML) and myelodysplastic syndromes (MDS) on NSABP B-25: an update (abstract 459). *Proc Am Soc Clin Oncol* **16**: 130A

Fisher RI, De Vita VT Jr and Hubbar SM (1983) Diffuse aggressive lymphomas; increased survival after alternating flexible sequences of ProMACE and MOPP chemotherapy. *Ann Intern Med* **98**: 304–309

Fisher RI, Gaynor ER, Dahlberg S, Oken MM, Grogan TM, Mize EM, Glick JH, Coltman CA and Miller TP (1993) Comparison of a standard regimen (CHOP) with three intensive chemotherapy regimens for advanced non-Hodgkin's lymphoma. *N Engl J Med* **328**: 1002–1006

Gordon LI, Andersen J, Habermann TM, Winter JN, Glick J, Schilder RJ and Cassileth P (1996) Phase I trial of dose escalation with Growth Factor support in patients with previously untreated diffuse aggressive lymphomas. Determination of the maximum tolerated dose of ProMACE-CytaBOM. *J Clin Oncol* **14**: 1275–1281

Gurney H, Dodwell D, Thatcher N and Tattersal MHN (1993*a*) Escalating drug delivery in cancer chemotherapy: a review of concepts and practice part 1. *Ann Oncol* **4**: 23–24

Gurney H, Dodwell D, Thatcher N and Tattersal MHN (1993*b*) Escalating drug delivery in cancer chemotherapy: a review of concepts and practice part 2. *Ann Oncol* **4**: 103–115

Hryniuk W (1994) Randomized trial of escalated vs standard BACOP (bleomycin, adriamycin, cyclofosfamide, oncovin, prednisone) for intermediate grade lymphoma. *Proc Am Soc Clin Oncol* **10**: 272

Hryniuk W and Bush H (1984) The importance of dose intensity in chemotherapy of metastatic breast cancer. *J Clin Oncol* **2**: 1281–1288

Kehoe S, Poole CJ, Stanley A, Earl HM and Blachledge GRP (1994) A phase I/II trial of recombinant human granulocyte–macrophage colony-stimulating factor in the intensification of cisplatin and cyclophosphamide chemotherapy for advanced ovarian cancer. *Br J Cancer* **69**: 537–540

Klimo P and Connors JM (1985) MACOP-B chemotherapy for the treatment of diffuse large-cell lymphoma. *Ann Int Med* **102**: 596–602

Kwak LW, Halpern J, Olshen RA and Horng SJ (1990) Prognostic significance of actual dose intensity in diffuse large-cell lymphoma: results of a tree structured survival analysis (1990). *J Clin Oncol* **8**: 963–977

Lalisang RI, Wils JA, Nortier HW, Burghouts JT, Hupperets PS, Erakamp FL, Schouten HC, Blijham GH (1997) Comparative study of dose escalation versus interval reduction to obtain dose-intensification of epirubicin and cyclophosphamide with granulocyte colony-stimulating factor in advanced breast cancer. *J Clin Oncol* **15**: 1367–1376

Lepage E, Gisselbrecht C, Haioun C, Sebban C, Hilly T, Bosly A, Morel P, Herbrecht R, Reyes F and Coiffier B, reporting for the GELA (1993) Prognostic significance of received dose intensity in non-Hodgkin's lymphoma patients: application to LNH-87 protocol. *Ann Oncol* **4**: 651–656

Longo DL, De Vita VT, Duffey PL (1991) Superiority of ProMACE-CytaBOM over ProMACE-MOPP in the treatment of advanced diffuse aggressive lymphoma: results of a prospective randomized trial. *J Clin Oncol* **9**: 25–38

Meyer RM, Hryniuk WM and Goodyear MDE (1991). The role of dose intensity in determining outcome in intermediate-grade non-Hodgkin's lymphoma. *J Clin Oncol* **9**: 339–347

Miller AB, Hoogstraten B, Staquet M and Winkler A (1981) Reporting results of cancer treatment. *Cancer* **47**: 207–214

Mouridsen HT, Alfthan C, Bashtolt L, Bergh M, Dalmark S, Eksborg S, Hellsten S, Kjaer M, Peterson C, Skovsgard T, Sorensen JB, Trope C and Aabo K (1990). Current status of epirubicin (farmorubicin) in the treatment of solid tumors. *Acta Oncol* **29**: 257–285

Pettengell R, Gurney H and Radford JA (1992) Dose limiting neutropenia in high grade non-Hodgkin's lymphoma: a randomised controlled trial of G-CSF. *Blood* **80**: 1430–1436

Pronzato P, Campora E, Amoroso D, Bertelli G, Botto F, Conte PF, Sertoli MR and Rosso R (1989) Impact of administration-related factors on outcome of adjuvant chemotherapy for primary breast cancer. *Am J Clin Oncol* **12**: 481–485

Pronzato P, Bertelli G, Vigani A and Vaira F (1996) A feasibility study of accelerated polychemotherapy with cisplatin, epidoxorubicin and cyclophosphamide (PEC) in advanced ovarian cancer. *Br J Cancer* **73**: 1425–1427

Pronzato P, Bertelli G, Bruna F, Tani F, Vaira F, Vanoli M and Vigani A (1997) Intensified M-VEC chemotherapy with G-CSF support as outpatient treatment for advanced bladder cancer. *Anticancer Res* **17**: 2325–2328

Shipp MA, Harrington DP and Klatt MM (1993) A predictive model for aggressive non-Hodgkin's lymphoma. *N Engl J Med* **329**: 987–994

Shipp MA, Neuberg D, Janiceck M, Cancellos GP and Shulman LN (1995) High dose CHOP as initial therapy for patients with poor prognosis aggressive non-Hodgkin's lymphoma: a dose finding pilot study. *J Clin Oncol* **13**: 2916–2923

Simon R (1989) Optimal two stage designs for phase II clinical trials. *Controlled Clin Trials* **10**: 1–10

© Cancer Research Campaign 1998